# Primary Intrathyroidal Non-Hodgkin Lymphoma: A Case Report

**DOI:** 10.7759/cureus.47096

**Published:** 2023-10-16

**Authors:** Kiril Hristozov, Radina Dimitrova, Savi Shishkov, Nadezhda Stefanova, Svetlana Gercheva

**Affiliations:** 1 Second Department of Internal Medicine, Medical University "Prof. Dr. Paraskev Stoyanov", Varna, BGR; 2 Department of General and Clinical Pathology, Forensic Medicine, and Deontology, Medical University "Prof. Dr. Paraskev Stoyanov", Varna, BGR

**Keywords:** hashimotos thyroiditis, case report, non-hodgkin lymphoma, primary intrathyroidal lymphoma, thyroid

## Abstract

Primary thyroid lymphoma (PTL) is a rare disease characterized by the appearance of a rapidly growing solid mass in the cervical region. A major risk factor is chronic autoimmune thyroiditis with lymphocytes infiltrating the thyroid gland. The lymphoproliferative disease is seen more frequently in the females. PTL usually develops in the sixth and seventh decades of life. We present a case of a 66-year-old woman with diffuse primary B-cell thyroid lymphoma with no prior evidence of underlying autoimmune thyroid pathology. The initial localization of the lymphoproliferative disease was in the thyroid gland, but the involvement of regional cervical lymph nodes was also found at the time of diagnosis. After histological verification with immunohistochemistry and staging by imaging, chemotherapy was initiated according to the R-CHOP (rituximab, cyclophosphamide, hydroxydaunorubicin hydrochloride, Oncovin^®^ (vincristine), prednisone) protocol. An excellent therapeutic response was achieved with lymphoma remission after six cycles under the mentioned protocol. Thyroid autoantibodies became positive 18 months after rituximab treatment, possibly reflecting the transient suppressive effects of the immunotherapy. The patient was subsequently kept followed up by a multidisciplinary team in the light of possible lymphoma recurrence and/or development of thyroid dysfunction. This case report demonstrates possible challenges for the diagnosis, treatment, and follow-up of this rare thyroid lesion. At the time of diagnosis, the clinical presentation of the disease, the ultrasound image, and the cytological result may be similar to other low-grade thyroid carcinomas or secondary metastatic involvement of the gland. The initial lack of underlying thyroid autoimmunity makes this distinction even more challenging. Furthermore, despite the rapid resolution, regular long-term monitoring for recurrence is required.

## Introduction

Thyroid lymphomas are primary or secondary depending on their location of occurrence. Primary thyroid lymphomas (PTL) first affect the thyroid gland and may subsequently involve distant lymph nodes and other organs. On the other hand, secondary lymphomas originate in another organ or lymph nodes and subsequently involve the thyroid gland [1]. PTL is generally a rare pathology. It accounts for about 5% of all malignant thyroid tumors and less than 2% of all extranodal lymphomas [2]. It usually develops based on underlying Hashimoto's thyroiditis, which increases the risk for PTL 40-80 fold. The incidence of chronic lymphocytic thyroiditis varies with different researchers and is reported to be 78.9% in a recent meta-analysis [3].

PTL typically presents in the sixth or seventh decade of life, usually in females [2]. The most common variant is diffuse B-cell lymphoma followed by mucosa-associated lymphoid tissue (MALT) lymphoma. Less frequent are T-cell lymphoma, NK-cell (natural killer cells) lymphoma, and Hodgkin lymphoma [4].

As a rule, the onset of PTL is associated with the appearance of a hard-growing lump in the cervical region. Unlike most types of thyroid neoplasia, thyroid lymphomas tend to grow very rapidly and cause compressive symptoms that include difficulty in swallowing, shortness of breath, and hoarse voice. Symptoms of B-cell lymphomas such as weight loss, fever, night sweats, and itching may also be observed. Furthermore, the presence of hypothyroidism is expected given the frequent association of PTL with underlying chronic lymphocytic thyroiditis.

The diagnosis of PTL is presumed based on the findings from the ultrasound examination; however, it is conclusively confirmed through cytological/histological verification. Computed tomography (CT), magnetic resonance imaging (MRI), or positron emission tomography (PET) are necessary for the subsequent staging of the disease.

The treatment of PTL depends on its histological variant and the disease stage. It usually involves chemotherapy and/or radiotherapy. Surgery is generally not recommended, except for performing a diagnostic biopsy, because of the potential surgical risks and lack of additional benefit [5].

## Case presentation

The presented case is of a 66-year-old woman admitted to the Endocrinology Clinic due to a growing lump in the cervical region accompanied by complaints of shortness of breath, pain in swallowing, and dysphonia with a duration of about one month. The clinical examination revealed an enlarged thyroid gland with a markedly firm consistency. Except for the compression by the cervical tumor mass, B symptoms like fever, weight loss, night sweats, or CNS involvement were not present at the time of diagnosis. 

Baseline hormonal investigations showed no significant thyroid function abnormalities apart from positive thyroid autoantibodies (Table 1). The patient had no family history of thyroid disease. No risk factors for lymphoma were identified including past or current bacterial and viral infections, exposure to chemicals, or ionizing radiation. 

**Table 1:** Thyroid function tests TSH: thyroid stimulating hormone; FT3: free triiodothyronine; FT4: free tetraiodothyronine; Anti-TPO: anti thyroperoxidase antibodies; Anti-TG: antithyroglobulin antibody

Parameter	Measurement unit and reference value	Baseline	Six months after chemotherapy	Seven months after chemotherapy	18 months after chemotherapy
TSH	0.27-4.2 µIU/ml	4.01	4.73	5.53	12.45
FT4	10.3-24 pmol/l	10.06	6.25	6.14	2.57
FT3	3.1-6.8 pmol/l	3.12	n/a	2.48	1.57
Anti-TPO	0-34 U/ml	34.3	13.8	15.4	15
Anti-TG	0-115 U/ml	622	n/a	16.0	1129

**Figure 1:**
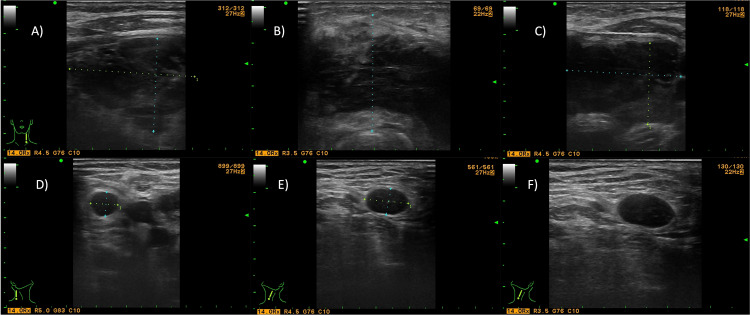
Ultrasonography at presentation Ultrasound images of the tumor formation, involving the right lobe (A), isthmus (B), and the left lobe (C) of the thyroid gland; images D, E, and F show pathological cervical lymph nodes.

The ultrasound revealed a hypoechoic formation involving both lobes and the isthmus of the thyroid gland. No normal thyroid parenchyma was visualized. Pathologically altered rounded lymph nodes with obliterated hilus were also found bilaterally in the cervical region (Figure 1).

Different neoplastic processes leading to the rapid growth of the thyroid gland such as anaplastic thyroid carcinoma, lymphoma, and metastatic invasion of the gland were discussed as differential diagnoses after ultrasonography.

**Figure 2:**
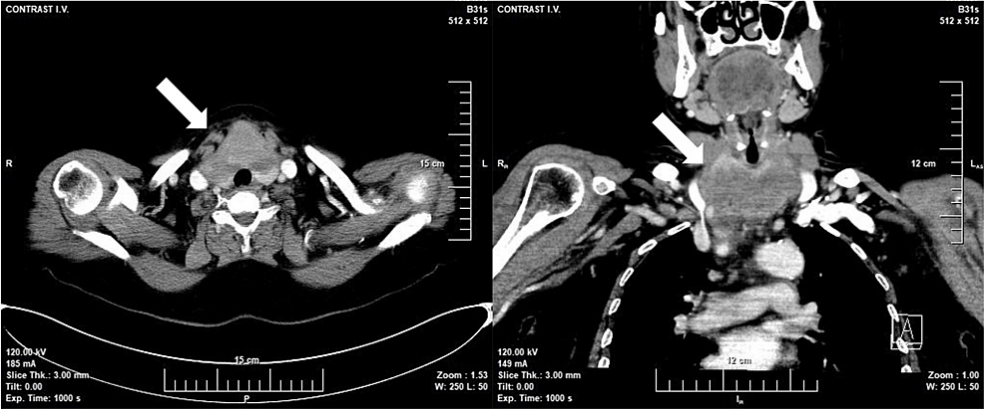
Neck CT at presentation showing axial and coronal sections of the tumor formation (arrow)

The additional CT scan of the neck region showed the presence of a 76/35/66 mm tumor in the thyroid region. The lesion extended to the sternoclavicular joints. It dislocated, slightly compressed, and stenosed the trunk vessels (Figure 2). Bilateral cervical lymphadenopathy was also visualized.

**Figure 3:**
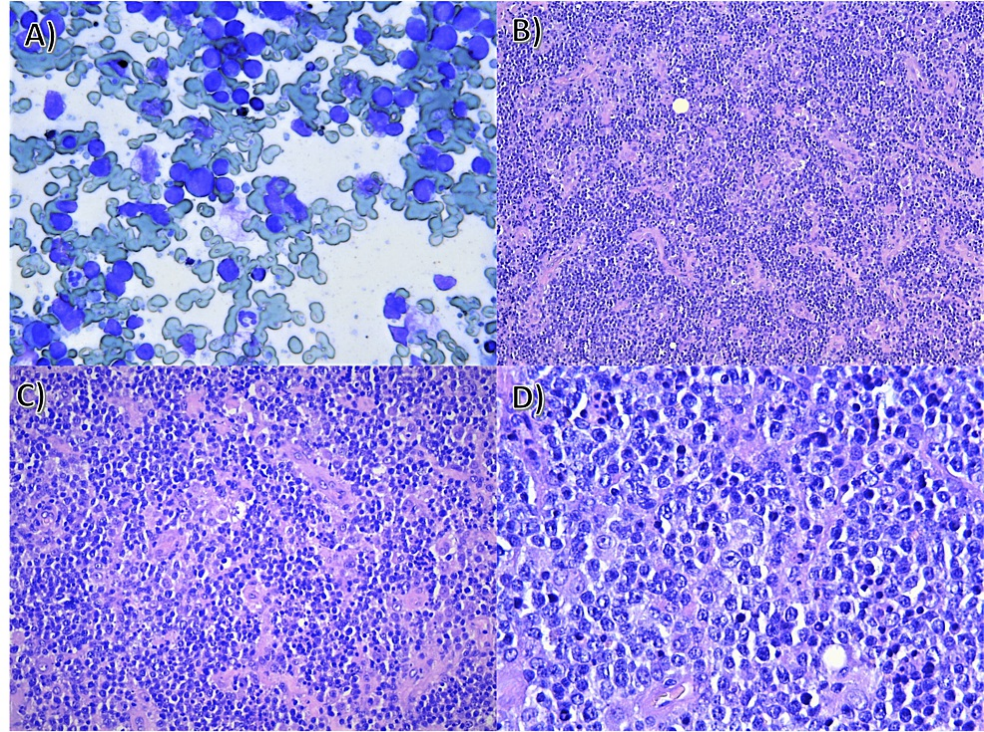
Cytological and histological findings (A) Scanty amount of colloid, pigmented macrophages, oncocytes, and large amount of atypical lymphoid cells that are large in size, elevated nuclear-cytoplasmic index, scanty cytoplasm, finely granular chromatin, and prominent nucleoli. Histology demonstrates complete effacement of the normal architecture, diffuse infiltrate of atypical lymphoid cells, magnification x400; (B) H&E stain, original magnification x100; (C) Diffuse monomorphic infiltrate of lymphoid cells separated by vascular proliferation, histiocytes, and collagen fibers, H&E stain, magnification x200; (D) Morphology of the lymphoid cells, large and medium-sized cells, some of the cells are with nuclear margination of the chromatin (centroblactic type), others with centrally placed nucleolus (immunoblastic type), H&E stain, magnification x400.

**Figure 4:**
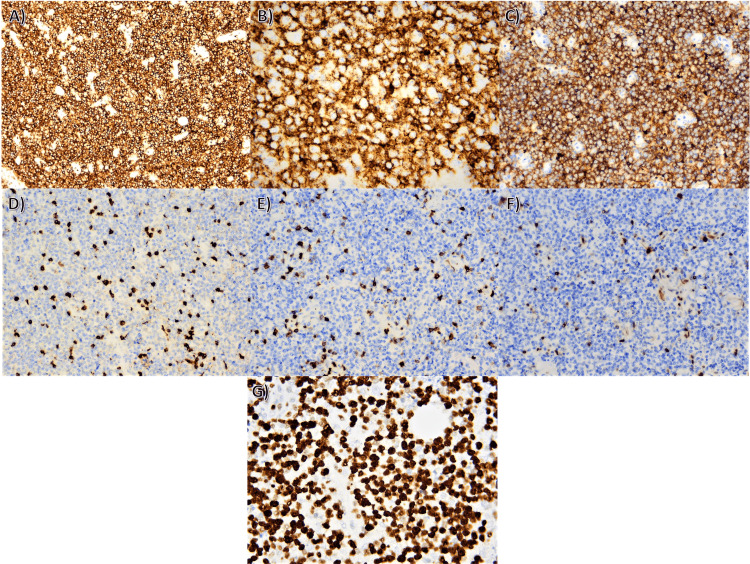
Immunohistochemical staining (A) Immunohistochemical stain with anti-CD45 antibody, diffuse positive expression in atypical cells, magnification x100; (B) Immunohistochemical stain with anti-CD20 antibody, diffuse positive expression in atypical lymphoid cells, magnification x400; (C) Immunohistochemical stain with anti-CD10 antibody, positive expression in the diffuse proliferating lymphoid cells, original magnification x200; (D) Immunohistochemical stain with anti-CD3 antibody, positive expression in small lymphocytes, negative expression in the tumor cells, original magnification x 200; (E) Immunohistochemical stain with anti-CD5 antibody, positive expression in small lymphocytes, negative expression in atypical cells, magnification x 200; (F) Immunohistochemical stain with anti-cyclin D1 antibody, negative expression in tumor cells, original magnification x 200; (G) Immunohistochemical stain with anti-Ki-67 antibody, positive nuclear expression in almost all of the atypical lymphoid cells, magnification  x 400.

Fine needle aspiration (FNA) biopsy of the tumor formation was performed for diagnostic verification. The cytological result confirmed a malignant process with morphology most similar to non-Hodgkin lymphoma (Figure 3A). For diagnostic confirmation, the patient subsequently underwent an incisional biopsy for histological verification (Figures 3B, 3C, 3D, 4). The result of the surgical intervention supported the diagnosis of non-Hodgkin's lymphoma. The histology demonstrated a completely obliterated structure by atypical lymphoid proliferation. The cells were medium and large lymphoid cells, most of them with submembranous nucleoli (centroblastic type), and some of them with central nucleoli (immunoblastic type). Scattered among them were single histiocytes, single and small groups of mature lymphocytes segregated by vascular proliferation and collagenized connective tissue bands. Immunohistochemical staining was positive for CD45, CD20, CD10, CD3, CD5, cyclin D1, and 100% Ki67 expression. The patient was subsequently referred to the Department of Hematology. Cell origin was not determined because genetic analysis was not available. 

A whole-body, contrast-enhanced CT scan was performed two months later for staging as PET-CT was unavailable at the time of diagnosis. It showed a progression in the size of the intrathyroidal formation (77/42/73 mm). No lesions in the chest and abdominal cavity or central nervous system were found. The performed trephine biopsy revealed no bone marrow infiltration. Based on laboratory, imaging, histological, and immunohistochemical investigations, the case was classified as stage IIB (Ann Arbor) diffuse B-cell lymphoma of undetermined classification (NOS) [5]. After the initial staging with contrast-enhanced CT, due to the localization of the tumor in the thyroid and neck region alone, no further imaging modality was considered necessary. No surgical debulking was undertaken due to the relatively mild compressive symptoms and lack of laryngeal nerve involvement. [6]

R-CHOP protocol (rituximab, cyclophosphamide, doxorubicin, vincristine, prednisolone) treatment was initiated with six cycles, repeated every 21 days, during the first six months after the diagnosis was made. This resulted in a rapid reduction in the size of the tumor formation as well as an improvement in the compressive symptoms. 

**Figure 5:**
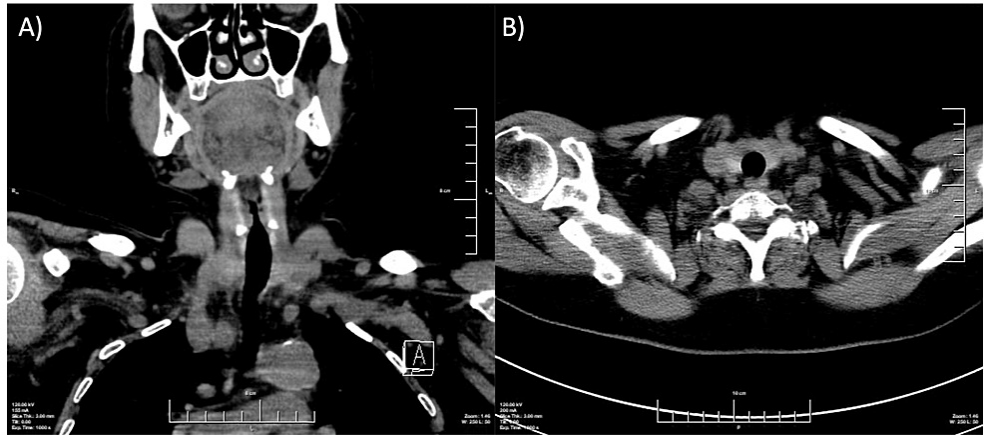
Post-chemotherapy CT scan CT image of the neck after six courses of chemotherapy. No tumor formation is present.

**Figure 6:**
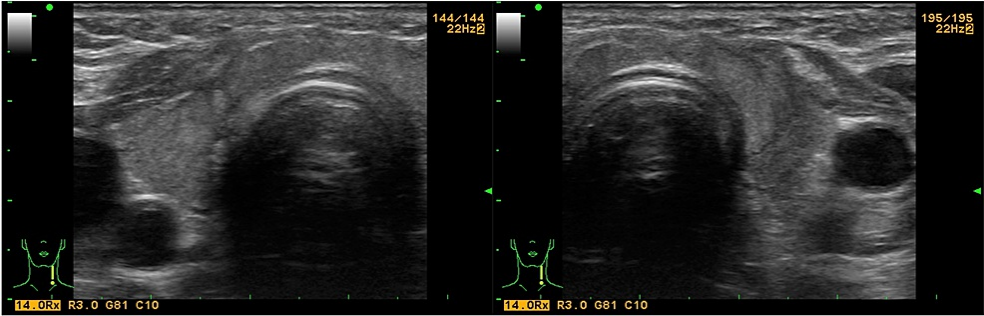
Ultrasound of the thyroid after six courses of chemotherapy The thyroid parenchyma has inhomogeneous structure. The formation seen at baseline is not visible.

A control whole-body CT scan on the sixth month after initiation of chemotherapy showed a complete reversal of the tumor formation in the thyroid region and no significant cervical lymphadenopathy (Figure 5). Therefore, radiotherapy was considered unnecessary. The absence of tumor formation was confirmed by follow-up ultrasonography, which revealed a heterogeneous image of the thyroid parenchyma in the absence of convincing evidence of autoimmune thyroid disease (Figure 6). Analysis of the monitored hormonal parameters indicated the development of hypothyroidism in the setting of negative thyroid autoantibodies (Table 1). 

**Figure 7:**
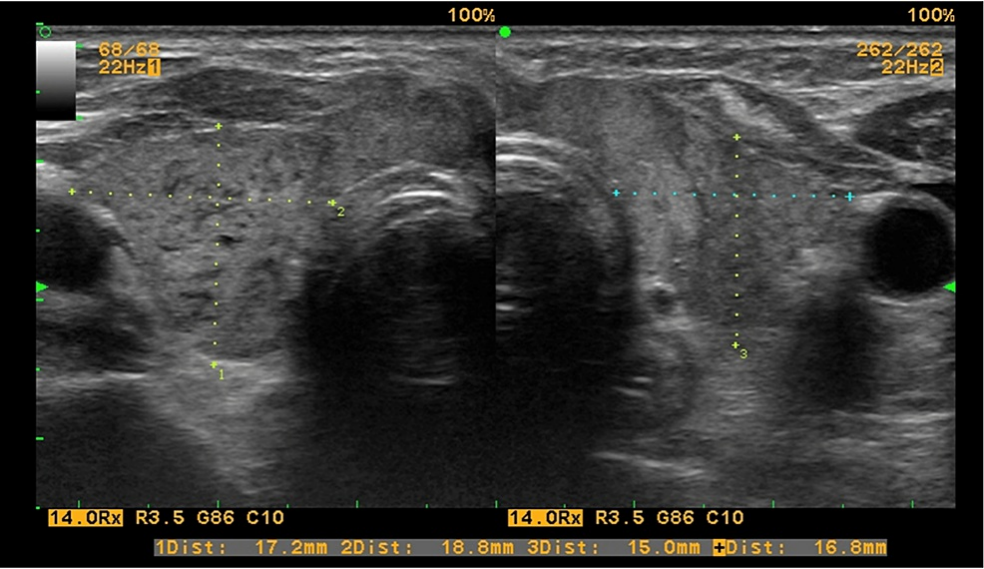
Ultrasound images of the thyroid one and a half years after the chemotherapy The thyroid gland hash slight heterogeneity of the parenchyma with small focal hypoechogenicity

One and a half years after the chemotherapy imaging, immunological data for autoimmune thyroiditis with overt hypothyroidism became evident (Table 1, Figure 7). Therefore, replacement therapy with levothyroxine was initiated. 

During an 18-month follow-up period, no recurrence of lymphoma was reported. The neck ultrasound examination revealed slight heterogeneity of the parenchyma with small focal hypoechogenicity. The patient continues levothyroxine replacement. Despite the achieved and sustained remission of lymphoproliferative disease, the patient should be periodically monitored by a multidisciplinary team for possible recurrence of lymphoma as well as for correction of abnormalities in thyroid function caused by underlying thyroiditis and/or chemotherapy. 

## Discussion

The risk of developing PTL is thought to be 40-80 fold higher in the presence of underlying Hashimoto's thyroiditis, as lymphocytic infiltration in the thyroid gland is associated with chronic antigenic stimulation, which is a trigger for malignant lymphoproliferative diseases [3]. On the other hand, only about 0.1% of the cases with Hashimoto's thyroiditis develop lymphoma [7].

In addition, at the time of diagnosis, the clinical presentation of PTC, the ultrasound image, and the cytological result may be similar to other low-grade thyroid carcinomas. The lack of known underlying autoimmunity at presentation makes this distinction even more challenging. Furthermore, despite the rapid resolution, regular long-term monitoring for recurrence is required.

In the presented case, there was no history of previous chronic lymphocytic thyroiditis. The initial positive titer of anti-thyroglobulin antibodies (anti-TG Ab) might be indicative of underlying thyroid autoimmunity but thyroid destruction as a consequence of malignant infiltration should also be considered. Strong ultrasonographic and immunological evidence of autoimmune thyroid disease immediately after chemotherapy was also lacking. However, a year and a half later, antithyroglobulin antibodies became positive and the ultrasonographic changes in the thyroid gland gave convincing evidence of Hashimoto's thyroiditis. A possible reason for the absence of thyroid antibodies soon after chemotherapy may be due to suppression by rituximab mediated by B-cell depletion [8,9].

Even in known lymphocytic thyroiditis, making the diagnosis of PLT in clinical practice can be challenging. The rapidly growing goiter, which is a hallmark of PTL, is not a specific manifestation and may be seen in other primary thyroid neoplasia as well as in secondary thyroid gland involvement by metastatic lesions. The ultrasound finding of PTL is also relatively nonspecific. It may be similar to other primary and secondary thyroid malignancies. This may further make the distinction between PTL and metastatic lesions or anaplastic thyroid carcinoma based on ultrasound image alone even more challenging. However, the ultrasound findings in the present case point to PTL, as the heterogeneous ultrasound structure from the lymphoproliferative infiltrates is not consistent with the presence of calcifications and areas of cystic degeneration in the thyroid gland.

On the other hand, cytological examination is one of the main methods to differentiate a malignant process from Hashimoto's thyroiditis. The lack of marked atypia in PTL makes a definitive cytological diagnosis difficult [10]. In contrast to other cases, FNA gave sufficient information to determine the tumor type, even without initial immunohistochemical staining [11]. However, histological verification was also performed to confirm the diagnosis.

In addition to the diagnostic challenges, this rare disease presents some challenges in terms of management and follow-up. Distinguishing the type of lymphoma and its stage of development is essential to determine the therapeutic approach and prognosis of the disease. Surgical intervention usually appears to be unnecessary as PTL has the potential to dramatically decrease in size over the course of chemotherapy and/or radiotherapy. However, surgical intervention for decompression and airway clearance may be indicated in certain cases [12]. In some studies, with a small number of patients, initial surgery has not led to prognostic advantage [13,14] in contrast to others where surgery is associated with better survival [15].

The presented case demonstrated an excellent response to the R-CHOP protocol. In this situation, surgical debulking was considered unnecessary. Invasive procedures were limited to biopsies, which were diagnostically indispensable. Despite the lack of PET-CT at diagnosis or after chemotherapy, the contrast-enhanced CT scan demonstrated improvement. Radiotherapy was also considered unnecessary due to the rapid reduction of the tumor size.

The follow-up of PTL is another ongoing issue as relapse is a real possibility. Despite the advanced age, which is associated with a poorer prognosis, a few other favorable factors could be mentioned as the relatively small size of the primary tumor (<10cm) and the lack of B-symptoms. [16]. In the described case, the follow-up continues twice a year with an ultrasound, hormonal, and immunological evaluations. A limitation of our case report is the short duration of follow-up (18 months), as future disease relapse is possible.

## Conclusions

PTL is a rare condition requiring a complex multidisciplinary diagnostic and therapeutic approach. The presented case demonstrated possible challenges for the diagnosis, treatment, and follow-up of this rare thyroid disorder. The disease should be thought of in any patient with compressive symptoms, enlarged lymph nodes, hoarse voice, and/or a rapidly growing mass in the neck. Timely diagnosis is essential for the treatment and prognosis of the disease.
